# Editorial: Sustainable strategies for the management of phytoparasitic nematodes

**DOI:** 10.3389/fpls.2023.1148261

**Published:** 2023-01-31

**Authors:** Carla Maleita, Ivânia Esteves, Aurelio Ciancio, Yuji Oka

**Affiliations:** ^1^Department of Chemical Engineering, University of Coimbra, Chemical Process Engineering and Forest Products Research Centre, Coimbra, Portugal; ^2^Department of Life Sciences, University of Coimbra, Centre for Functional Ecology - Science for People & the Planet, Coimbra, Portugal; ^3^Istituto per la Protezione Sostenibile delle Piante, Consiglio Nazionale delle Ricerche, Bari, Italy; ^4^Nematology Unit, Gilat Research Center, Agricultural Research Organization, M. P. Negev, Israel

**Keywords:** biocontrol agents, bionematicides, cultural methods, *Meloidogyne* spp., *Nacobbus* spp., nematode control, plant-parasitic nematodes, resistance

Plant-parasitic nematodes (PPN), “the unseen enemies” of plants, are a threat to economically important crops, affecting production, quality, and yields, with losses estimated at 173 billion US dollars/year (Elling, 2013). The top 10 PPN, based on their scientific and economic importance, include root-knot (RKN, *Meloidogyne* spp.), cyst (*Heterodera*/*Globodera* spp.), and root-lesion nematodes (RLN, *Pratylenchus* spp.), followed by other species.

For the last 50 years, PPN control has relied on the use of synthetic nematicides and soil fumigants that are rapid-acting, and specifically reliable. Nevertheless, due to environmental concerns and human health issues, most traditional nematicides have been exposed to an increasing regulatory pressure, with many products banned or withdrawn from the market. Nowadays, the adoption of control strategies based on the use of safer and selective bionematicides, biocontrol agents, cultural methods, and plant resistance is highly advisable, in order to achieve sustainable PPN control. However, some of these strategies, and their integration, are not always available or appear less consistent, compared to synthetic nematicides. In particular, microbial antagonists are selected to improve efficacy of bioformulations *vs* PPN, through direct application or active metabolites. However, the rhizosphere is a complex system including thousands of microbial species, whose effect is known only in part. A schematic representation of such tri-trophic (plant-nematode-antagonists) food web is given in **Figure 1**.

The main goal of this topic was indeed to bring together recent advances in sustainable management strategies for important PPN. The contributions include potential bionematicides, evaluation of their efficacy and mode of action (Maleita et al.); exploitation of biocontrol bacteria (Singh and Wesemael; Diaz-Manzano et al.); and a review on *Nacobbus* spp. management (Lax et al).

The effects of two natural compounds (juglone and 1,4-naphthoquinone), which can be extracted from walnut husk residues, were evaluated on the *Meloidogyne luci* life cycle (Maleita et al.). This RKN has been detected in several countries in Europe, associated to economically important crops. Although there are no data available on *M. luci* impacts, host suitability assays suggested that significant losses can be induced by this species (Maleita et al., 2018; Aydinli et al., 2019; Sen and Aydinli, 2021; Maleita et al., 2022). For this reason, *M. luci* has been included since 2017 in the Alert List of Pests of the European and Mediterranean Plant Protection Organization (EPPO, 2017; EPPO, 2021). Results showed that both compounds increased the *in vitro* mortality of the second-stage juveniles (J2) and reduced hatching by ≈50%. In pot assays with tomato plants, J2 infection was also reduced by ≈80%. Both compounds are thus promising in the development of novel, natural and effective bionematicides, contributing to limiting crop damage caused by RKN. Finally, the mode of action of both natural compounds was also tentatively inferred through the assessment of reactive oxygen species (ROS) generation, *in vitro* inhibitory activity of acetylcholinesterase, and gene expression analysis. Since only 1,4-naphthoquinone induced the formation of ROS, the authors conclude that the action modes of the compounds are probably different. Studies on the *M. luci* transcriptome will be needed to provide further insights on the nematode metabolism and the nematicidal mechanisms reported.

**Figure 1:**
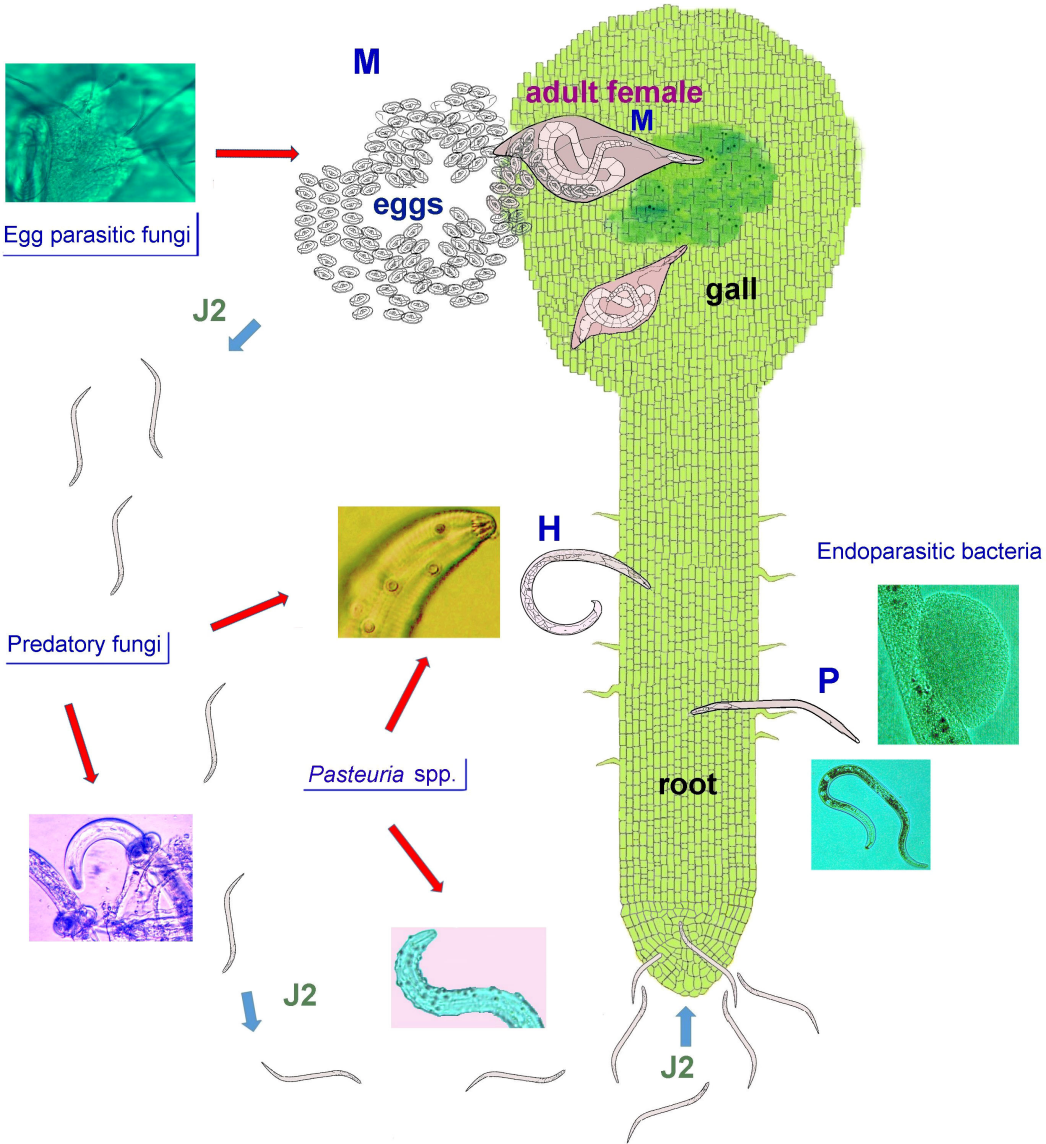
A schematic representation of a generic tri-trophic food web showing most common types of plant-parasitic nematode (PPN) antagonists, occurring in the rhizosphere environment. Three PPN with different lifestyles and trophic behaviour are shown: a sedentary root-knot nematode (RKN, M), *Meloidogyne sp.*; a migratory ectoparasite, such as the spiral nematode *Helicotylenchus* sp. (H), and a migratory endoparasite, *Pratylenchus* sp. (P). A number of fungi parasitize RKN eggs in soil or in colonized egg masses. The RKN second stage juveniles (J2), emerging from the remaining healthy eggs, migrate in soil toward the root apex. During this limited time period the J2 may encounter traps of other predatory fungi and/or infective propagules of bacterial endoparasites, i.e. *Pasteuria* spp. Once inside the roots, the J2 induce histological alterations yielding a root swelling (gall) with a feeding site, allowing the immature females development and adults reproduction. At this stage *Pasteuria* spp. complete their life-cycle in synchronism with the host and digest its body content, before being released in the rhizosphere environment (not shown). Also ecto- and endoparasitic nematodes are exposed in soil to fungal predators and/or to infection by bacteria, including other host-specific *Pasteuria* spp. and several unclassified species.

Singh and Wesemael and Díaz-Manzano et al. focused on bacterial biocontrol agents (*Paenibacillus polymyxa*, *Bacillus paralicheniformi* and *B. subtilis*) against RKN. The gram-positive, rod-shaped bacterium *Paenibacillus polymyxa* showed biological activity against different organisms, including bacteria, fungi, oomycetes, and PPN (Khan et al., 2008; Son et al., 2009; Cheng et al., 2017; Chávez-Ramírez et al., 2020; Chávez-Ramírez et al., 2021). Singh and Wesemael evaluated the dose-response effect of *P. polymyxa* on tomato susceptibility to *M. incognita* and plant growth. Lower numbers of second/third/fourth juveniles, galls, and females were observed in tomato roots treated with increasing concentrations of the bacterium. Tomato plants treated with different concentrations of *P. polymyxa*, with/without nematodes, showed a dose-dependent effect on growth. A negative effect was observed on root/shoot parameters, but it decreased in presence of *M. incognita*, suggesting a strong activation of plant defenses against the nematode attack. *Paenibacillus polymyxa* also showed a nematicidal activity inhibiting hatching, increasing the nematode mortality at the highest concentration tested. The authors hence suggest that the application of *P. polymyxa* as soil drenching, at seeding/planting, and at the end of the nematode life cycle (egg masses formation), may protect the host from nematodes penetration, and prevent hatching.

Díaz-Manzano et al. identified a potential nematode biological control strategy based on the combination of *B. paralicheniformi* and *B. subtilis*. The dual strain combination affected RKN hatching, survival, penetration and reproduction and RLN parasitism in soybean.

A further paper reviewed current insights into the biology, economic importance, and management of the false root-knot nematodes *Nacobbus* spp. (Lax et al.). Nematodes of this genus, native to the American continent, are important sedentary endoparasites responsible for significant economic losses on main food crops. However, the lack of knowledge on their life cycle, real impact on crops, and reliable methods of identification/detection made their management difficult. Lax et al. presented the latest outcomes on eco-friendly and sustainable management strategies of *Nacobbus* spp. They include the use of bacteria, entomopathogenic nematodes and their symbiotic bacteria, mycorrhizal and nematophagous fungi, essential oils, plant extracts, phytohormones, amendments, and plant resistance. The combined use and synergistic effects of the different strategies mentioned above were also discussed, together with strategies, such as crop rotation, nematicides, amendments, and biological control, biofumigation combined with arbuscular mycorrhizal fungi (AMF), as well as resistance, grafting and AMF symbiosis.

In many countries, the application of synthetic nematicides is still the most frequent strategy adopted by farmers for PPN control. Nevertheless, the concern for environmental protection and related issues arose not only within the scientific community but also among industry, farmers, and the society. This progressive concern is promoting the search for sustainable management strategies for important PPN. Several approaches have been developed, but their application is not ubiquitous, as many variables affect the soil food webs, related to climate, soil microbiome and texture, plant genotypes, and technical or agronomic specificities. Further progress is needed on many issues, including trainings of farmers and professionals, for the correct application of management practices and bioformulations. Main goal is also preventing the emergence of virulent populations and/or the insurgence of other PPN species that often coexist in the rhizosphere environment, at low density levels.

## Author contributions

CM, IE, AC and YO contributed to conception of the Editorial. CM wrote the first draft of the manuscript. All authors contributed to manuscript revision, read, and approved the submitted version.
